# *USH2A* Mutation is Associated With Tumor Mutation Burden and Antitumor Immunity in Patients With Colon Adenocarcinoma

**DOI:** 10.3389/fgene.2021.762160

**Published:** 2021-11-02

**Authors:** Yuanyuan Sun, Long Li, Wenchao Yao, Xuxu Liu, Yang Yang, Biao Ma, Dongbo Xue

**Affiliations:** Laboratory of Hepatosplenic Surgery, Department of General Surgery, Ministry of Education, The First Affiliated Hospital of Harbin Medical University, Harbin, China

**Keywords:** colon adenocarcinoma, *USH2A*, tumor mutation burden, immunotherapy response, bioinformatics analysis

## Abstract

Colon adenocarcinoma (COAD) is one of the diseases with the highest morbidity and mortality in the world. At present, immunotherapy has become a valuable method for the treatment of COAD. Tumor mutational burden (TMB) is considered to be the most common biomarker for predicting immunotherapy. According to reports, the mutation rate of COAD ranks third. However, whether these gene mutations are related to TMB and immune response is still unknown. Here, COAD somatic mutation data were downloaded from The Cancer Genome Atlas (TCGA) and International Cancer Genome Consortium (ICGC) databases. Bioinformatics methods were used to study the relationships among gene mutations, COAD survival prognosis, and tumor immune response. A total of 22 of the top 40 mutations in TCGA and ICGC databases were the same. Among them, the *USH2A* mutation was associated with high TMB and poor clinical prognosis. According to Gene Set Enrichment Analysis (GSEA) and the CIBERSORT algorithm, we determined that the *USH2A* mutation upregulates signaling pathways involved in the immune system and the antitumor immune response. In cases with a *USH2A* mutation, the immune score and MSI score of TCGA samples increased, the expression of immune checkpoint genes decreased significantly, and the TIDE score decreased significantly. Dependent on the presence or absence of a *USH2A* mutation, TCGA COAD samples were analyzed for differentially expressed genes, 522 of which were identified. Using a univariate Cox analysis and LASSO COX analysis of these differential genes, a prediction model was established, which established significant differences in the infiltration of immune cells, immune checkpoint gene expression, immune score, MSI score, TMB, and TIDE in patients in high- and low-risk groups. In conclusion, mutation of *USH2A* is frequent in COAD and is related to an increase in TMB and the antitumor immunity. The differential genes screened by *USH2A* mutation allowed the construction of a risk model for predicting the survival and prognosis of cancer patients, in addition to providing new ideas for COAD immunotherapy.

## 1 Introduction

Colon cancer is the third leading cause of cancer deaths, with more than one million new cases diagnosed each year (Labianca et al., 2010). COAD is the main pathological type of colon cancer. The incidence of COAD is mainly related to age and eating habits, and partly related to genetic diseases (Cunningham et al., 2010; Watson and Collins, 2011). COAD is heterogeneous, and there are significant differences in mutation patterns across different patients (Punt et al., 2017). Increasing evidence has shown that COAD is a molecular heterogeneous disease that contains a series of genetic changes (Choi et al., 2015). Mutations in key genes can affect tumor cell proliferation, differentiation, apoptosis, viability, and distant metastasis (The Cancer Genome Atlas Network, 2012). Surgery combined with postoperative chemotherapy is currently the main treatment for COAD. Although current treatment methods including chemotherapy and surgery have improved the survival rate of COAD patients, the prognosis of COAD patients is still poor (Neri et al., 2010; Roncucci and Mariani, 2015). The use of reliable biomarkers and the timely diagnosis of treatment targets can significantly improve the mortality of COAD patients and reduce the incidence of COAD (Herzig and Tsikitis, 2015; Tsimberidou, 2015). The immune system plays an important role in the occurrence and development of cancer (Patel and Minn, 2018). The 2020 ESMO clinical practice guidelines for colon cancer recommend the use of immune scores to improve the prognosis of colon cancer (Argilés et al., 2020). Therefore, it is necessary to study the relationship between specific genetic variants and immune events, as well as alternative methods of treating patients with different genetic characteristics. The accumulation of somatic mutations is one of the main causes of tumors and contributes to the expression of neoantigens (Gubin et al., 2015). Studies have shown that TMB is correlated with immunotherapy response (Goodman et al., 2017). It was reported that a high TMB can predict the prognosis of non-small-cell lung cancer and melanoma (Chen et al., 2019a; Chen et al., 2019b). Furthermore, TMB is considered to be a predictive biomarker of tumor behavior and immune response (Goodman et al., 2017).

Immune checkpoint blocking therapy (ICB), which targets programmed cell death ligand 1 (*PDL1*) and cytotoxic T lymphocyte antigen 4 (*CTLA4*) pathways, has become a treatment strategy for various types of cancer (Long et al., 2017; Zhang et al., 2021). TMB is an indicator that is independent of the expression level of *PDL1* and can better indicate the response to ICB treatment (Hodges et al., 2017; Rizvi et al., 2018). A comprehensive analysis of 27 cancer types reported that TMB is associated with better ICB treatment effects (Yarchoan et al., 2017). At present, the proportion of patients benefiting from ICB treatment in clinical practice is still very low, and new biomarkers that predict the ICB response rate of patients need to be developed (Ancevski Hunter et al., 2018; Janjigian et al., 2018). The Tumor Immune Dysfunction and Exclusion (TIDE) algorithm is a calculation method that uses gene expression profiles to predict the ICB response in non-small-cell lung cancer and melanoma (Jiang et al., 2018). TIDE uses a set of gene expression markers to estimate two different mechanisms of tumor immune evasion, including tumor-infiltrating cytotoxic T lymphocyte (CTL) dysfunction and immunosuppressive factor rejection of CTL. A higher TIDE score denotes a higher chance of antitumor immune escape and a lower response rate of ICB therapy (Jiang et al., 2018). TIDE score is more accurate than *PDL1* expression level and TMB in predicting the survival and prognosis of cancer patients treated with ICB (Jiang et al., 2018; Kaderbhaï et al., 2019; Keenan et al., 2019; Wang et al., 2019b). Several recent studies have reported its use in predicting or evaluating the effects of ICB treatment (Bretz et al., 2019; George et al., 2019; Liu et al., 2019; Pallocca et al., 2019; Wang et al., 2019b). At present, whether gene mutations are related to the COAD immune response and ICB treatment response remains unclear.

In this study, we used The Cancer Genome Atlas (TCGA) and the International Cancer Genome Consortium (ICGC) databases to identify somatic mutations in COAD patients in the United States and China. Then, we identified common mutant genes in both cohorts, which were found to be related to TMB and prognosis, thus confirming that gene mutations are related to immune response and ICB treatment response. On the basis of their differential expression caused by mutations, we constructed a prognostic model composed of two genes with a predictive effect on tumor prognosis and ICB treatment response. These findings reveal that a gene mutation can be used as a biomarker for predicting immune response and for evaluating the response to ICB treatment in patients with COAD.

## 2 Materials and Methods

### 2.1 Data Collection

**FIGURE 1:**
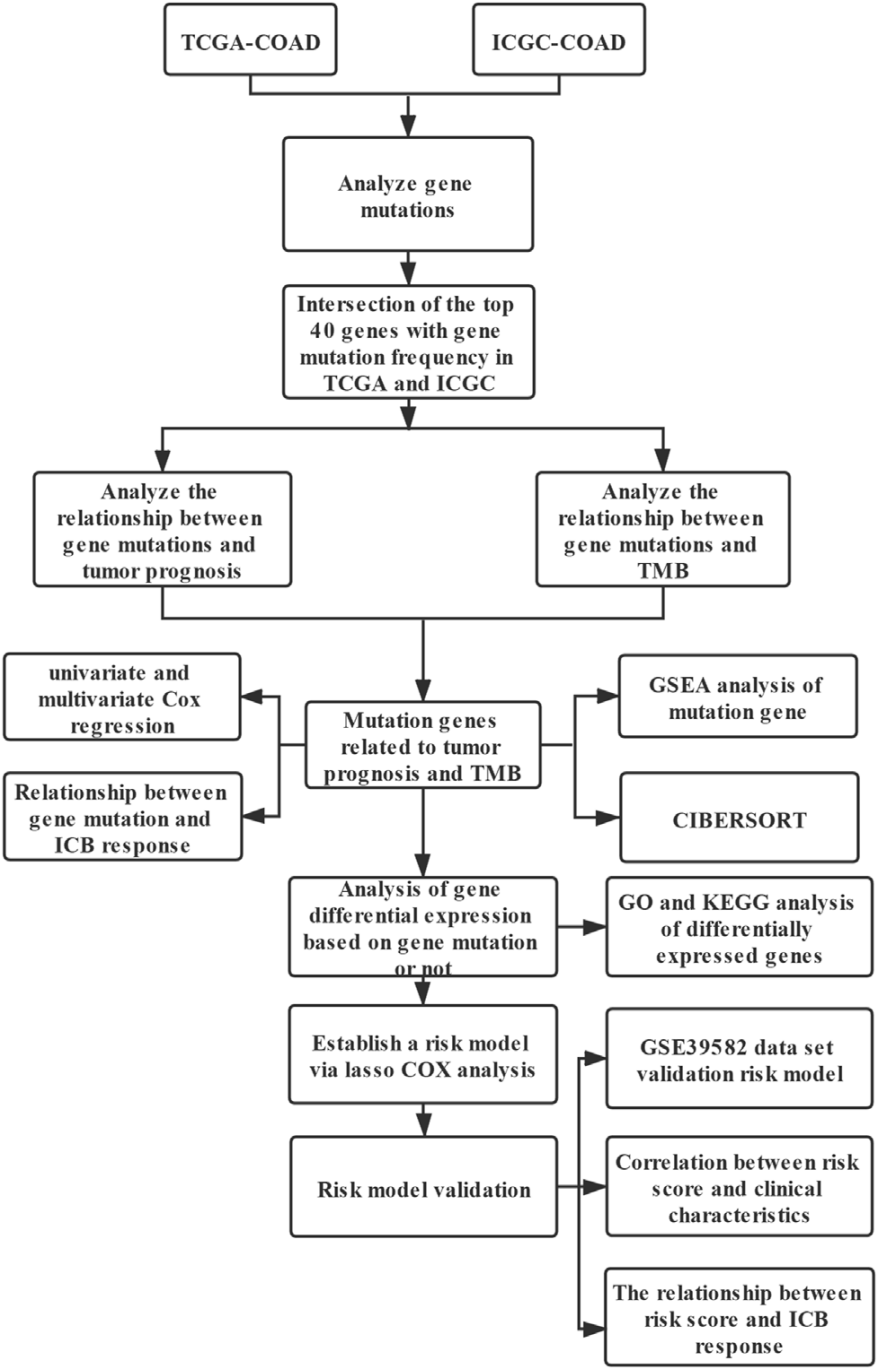
Analysis flow chart.

We used a method similar to that of Gongmin Zhu et al (2020). As shown in the flowchart (Figure 1), we downloaded transcriptome data (*n* = 444), clinical data (*n* = 336), and somatic gene mutation data (*n* = 398) from TCGA database (http://portal.gdc.cancer.gov/projects) (data updated on 29 October 2020). For clinical data, patients with COAD were included only when their clinical information was complete, and patients without survival time, survival status, age, gender, grade, or TNM classification data were not included. Next, the somatic gene mutation data of Chinese COAD patients (*n* = 305) was downloaded from the ICGC database (http://dcc.icgc.org/releases/current/Projects) (data updated on 27 November 2019), and the COAD dataset GSE39582 was downloaded from the GEO database (https://www.ncbi.nlm.nih.gov/geo/).

### 2.2 Bioinformatics Analysis

PERL software (version 5.32) was used to extract and sort TCGA transcription data, somatic mutation data, clinical data, ICGC mutation data, and GEO transcription data for subsequent analysis. R software (version 4.0.3) package GenVisR was used to analyze and visualize the MAF file of Varscn, the somatic mutation data of colon cancer in the TCGA database. R software package GenVisR was used to analyze and visualize the colon cancer somatic mutation data TSV file of the ICGC database based on the hg19 genome reference information. The venn package in R software was used to take the intersection of the top 40 genes in the TCGA and ICGC datasets with mutation evaluation rates, followed by obtaining the intersection genes with the top mutation frequencies in both databases. Next, R software package ggpubr was used to analyze the relationship between gene mutations and TMB. The Kaplan–Meier (KM) method was used to analyze the relationship between gene mutation and survival prognosis. Univariate and multivariate Cox methods were used to analyze the relationships among patient clinical information (age, gender, tumor stage, and TNM classification), TMB, gene mutations, and tumor survival prognosis. In all comparisons, a *p*-value < 0.05 was considered statistically significant. Software GSEA (version 4.1.0) was used for gene enrichment analysis. According to the gene mutations, TCGA expression data were divided into two groups: mutation and wild-type. The arrangement was set to 1,000, and the standardized enrichment score (NES) was applied using an FDR *q*-value < 0.05 as the significance threshold for enrichment (Subramanian et al., 2005). The edge R package was used to analyze the differentially expressed genes between the gene mutant group and the unmutated group (wild-type group). In the analysis process, genes were considered significantly differentially expressed for a *p-*value < 0.05 and a fold-change (FC) difference >2 (i.e., absolute value of log_2_ FC > 1). The enrichment analysis tool DAVID (Da et al., 2009) was used to analyze the Gene Ontology (GO) (Ashburner et al., 2000)functions and KEGG (Minoru and Susumu, 2000) pathways involved in upregulated and downregulated genes (number of parameter-enriched genes ≥2, *p-*value of hypergeometric test <0.05). R software was used to perform KM survival analysis and univariate Cox analysis of the differential genes using a *p*-value < 0.05 as the filter value. R software package glmnet was used to perform LASSO COX regression analysis and construct a prognosis-related risk model. To evaluate the risk model, R software package survival ROC was used to analyze the prediction accuracy of the model, and univariate and multivariate Cox analyses were used to evaluate whether the risk score of the tumor patient model could be used as an independent prognostic factor.

### 2.3 Tumor Mutation Burden and Evaluation of Microsatellite Instability

Tumor mutation burden (TMB) refers to the total number of gene coding errors, base substitutions, and gene insertion or deletion errors per megabit (Mb) of tumor tissue. All base substitutions and insertions in the coding region of the target gene are counted, whereas silent mutations that cannot cause amino-acid changes are not counted. The total number of mutations counted was divided by the exome size (the estimated value of the exome size was 38 Mb) to calculate the TMB score for each sample (Chalmers et al., 2017). A microsatellite is defined as a region of 10–60 base pairs containing 1–5 repeated base-pair motifs (Shia, 2015). Nucleotides in repetitive DNA fragments are spontaneously lost or repeated to form microsatellite instabilities (MSIs) (de la Chapelle and Hampel, 2010). According to the methods of Bonneville et al., the MSI score of COAD samples was characterized (Bonneville et al., 2017).

### 2.4 Tumor Immune Cell Infiltration Analysis

CIBERSORT is a deconvolution algorithm that can evaluate the proportion of 22 tumor-infiltrating lymphocyte subsets in a large number of tumor samples (Newman et al., 2015). This algorithm is used to evaluate the relative abundance of immune cell infiltration in tumor tissues. The number of permutations was set to 1,000, and a *p*-value < 0.05 was used as the basis for the successful calculation of the sample.

### 2.5 Prediction of ICB Treatment Response

R package estimate was used to calculate the immune score of the tumor sample. The Tumor Immune Dysfunction and Exclusion (TIDE) algorithm is a calculation method that uses gene expression profiles to predict immune checkpoint blockade (ICB) responses in non-small-cell lung cancer and melanoma (Jiang et al., 2018). Accordingly, it was used to predict the potential ICB response (Jiang et al., 2018).

### 2.6 Data Analysis

R software (version 4.0.3) was used for statistical analysis and graphing. The logrank test was used for KM survival analysis, and the Mann–Whitney U test was used for analysis of the relationship between gene mutation and TMB. In all comparisons, a *p*-value < 0.05 was considered statistically significant.

## 3 Results

### 3.1 COAD Somatic Mutations

**FIGURE 2:**
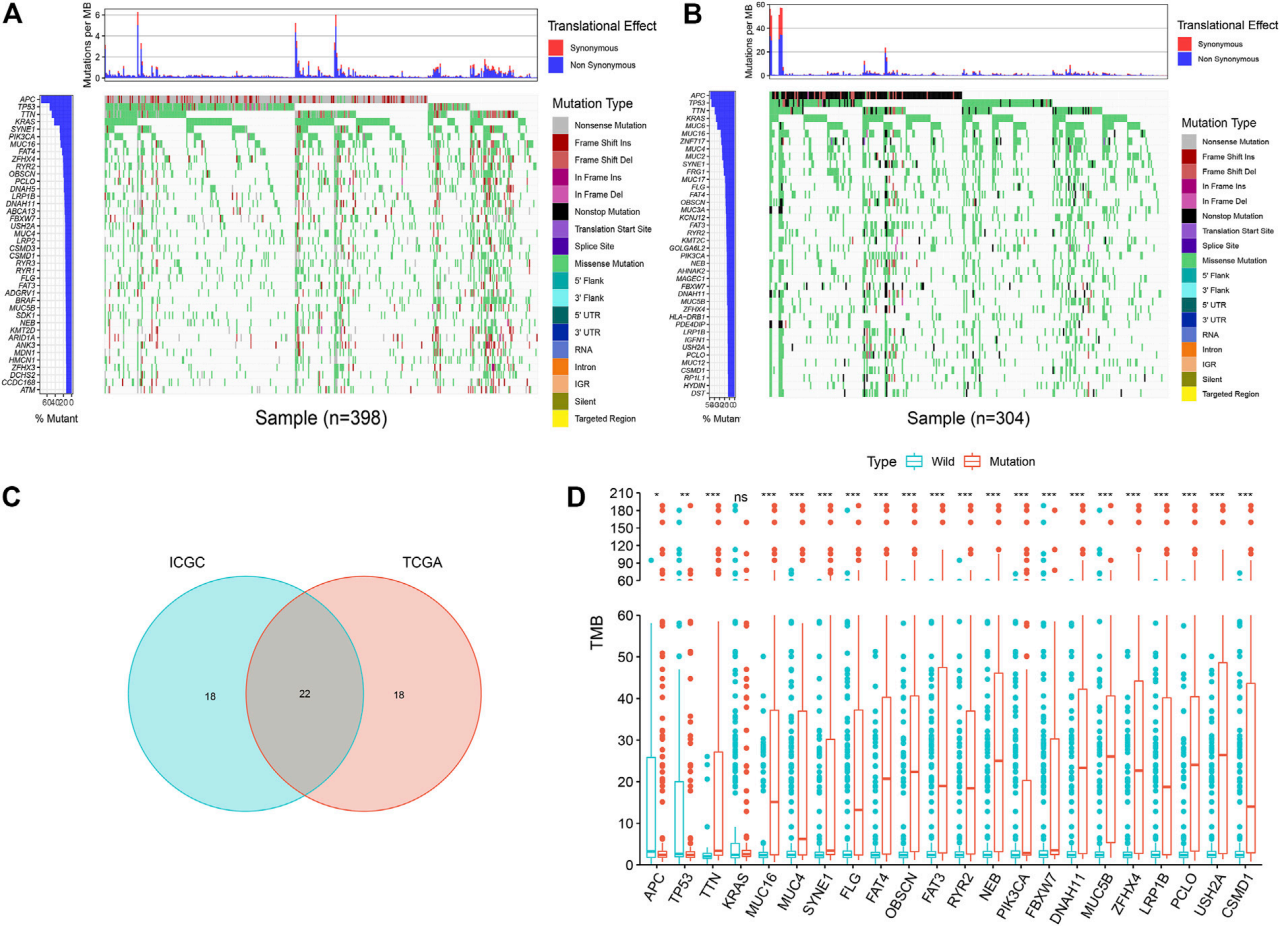
Overview of frequently mutated genes and TMB in COAD. **(A)** Waterfall plot shows the frequently mutated genes in COAD from TCGA database. The left panel shows mutation frequency, and genes are ordered by their mutation frequencies. The right panel presents different mutation types. **(B)** Waterfall plot displaying the frequently mutated genes in COAD from the ICGC cohort. The left panel shows the genes ordered by their mutation frequencies. The right panel presents different mutation types. **(C)** Venn diagram shows 22 frequently mutated genes covered by both the TCGA and ICGC cohorts. **(D)** 21 genes with high mutation frequency are associated with a higher TMB. ***p < 0.001, **p < 0.01, *p < 0.05, ns has no statistical difference.

The analysis found that, in the mutation data of TCGA samples, the top five most frequently mutated genes were *APC*, *TP53*, *TTN*, *KRAS*, and *SYNE1* (Figure 2A). In the mutation data of ICGC samples, the top five most frequently mutated genes were *APC*, *TP53*, *TTN*, *KRAS*, and *MUC6* (Figure 2B); thus, we identified some genes with high mutation frequencies in both databases. Therefore, we selected the top 40 genes in both databases, whereby we found an overlap of 22 genes, as depicted in a Venn diagram (Figure 2C).

### 3.2 *USH2A* Mutation is Associated With Tumor Mutation Burden and Survival Prognosis

**FIGURE 3:**
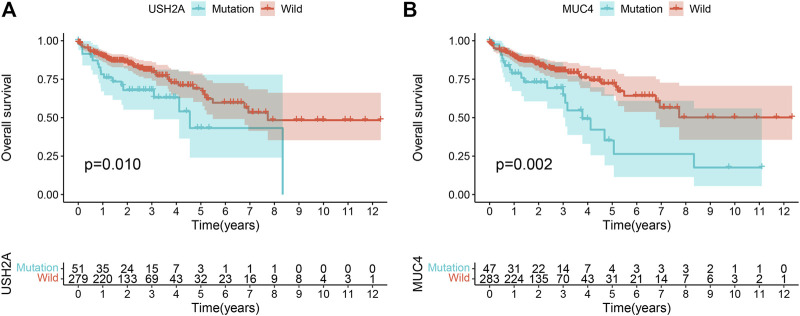
Gene mutation is associated with clinical prognosis. Kaplan-Meier survival analysis was used to determine survival curves that reflect the association between gene mutations and prognosis. The *p*-value is shown each plot. **(A)** USH2A mutation is associated with the prognosis of COAD. **(B)** MUC4 mutation is associated with the prognosis of COAD.

The mutation burden of COAD ranges from 0.05 to 188.31/Mb, with a median of 2.45/Mb. Among the 22 genes screened by the Venn diagram, the mutation of 21 genes was statistically related to the tumor mutation burden in the sample (Figure 2D). In order to study the relationship between these gene mutations related to tumor mutation burden and the prognosis of COAD, we further performed Kaplan–Meier analysis. The calculation results of KM analysis (Table 2) showed that the *USH2A* mutation and *MUC4* mutation were related to tumor survival and prognosis (Figure 3). Next, the mutation gene and the tumor patient’s age, gender, tumor stage, and tumor mutation burden were analyzed by univariate and multivariate Cox regression. The results (Table 1) showed that the *USH2A* mutation (HR = 1.909; 95% CI = 1.088–3.351; *p* = 0.024) and *MUC4* mutation (HR = 2.232; 95% CI = 1.301–3.829; *p* = 0.004) were associated with a poor prognosis of COAD; thus, they could be considered independent risk factors. We further studied the relationship between the location of the USH2A mutation site in the COAD sample of the TCGA database and the survival of COAD. We searched the UCSC database (http://genome.ucsc.edu/, hg38) and found that the mutation sites provided are distributed in the exon region of the USH2A gene (Supplementary Table S2). We analyzed the mutation regions with a sample size greater than 2 (exons 17, 61, 63, 64, 70) and found that the mutations located in exon 17 and exon 63 of USH2A are related to the survival of COAD (Supplementary Figure S2).

**TABLE 1:** Univariate and multivariate COX overall survival analysis of patients with COAD.

Factors	Univariate		Multivariate	
	HR(95% CI)	*p*-value	HR(95% CI)	*p*-value
Age (year) (≤65, >65)	1.829 (1.087–4.246)	0.023	2.475 (1.442–4.246)	0.001
Gender (male, female)	1.345 (1.087–4.246)	0.227		
Stage (I and II, III and IV)	2.831 (1.723–4.652)	<0.001	3.380 (2.022–5.647)	<0.001
TMB (low, high)	1.002 (0.991–1.012)	0.780		
MUC4	2.232 (1.301–3.829)	0.004	2.054 (1.196–3.528)	0.009
USH2A	1.909 (1.088–3.351)	0.024	2.067 (1.169–3.655)	0.012

**TABLE 2:** The clinical prognostic calculation results of gene mutations related to TMB.

Gene	*p*-value	Gene	*p*-value
MUC4	0.002	PIK3CA	0.466
USH2A	0.010	MUC5B	0.469
TTN	0.077	TP53	0.527
RYR2	0.151	FBXW7	0.616
NEB	0.154	MUC16	0.675
SYNE1	0.167	FAT3	0.772
LRP1B	0.222	APC	0.819
FLG	0.325	PCLO	0.863
ZFHX4	0.395	OBSCN	0.876
CSMD1	0.447		

### 3.3 Gene Set Enrichment Analysis

**FIGURE 4:**
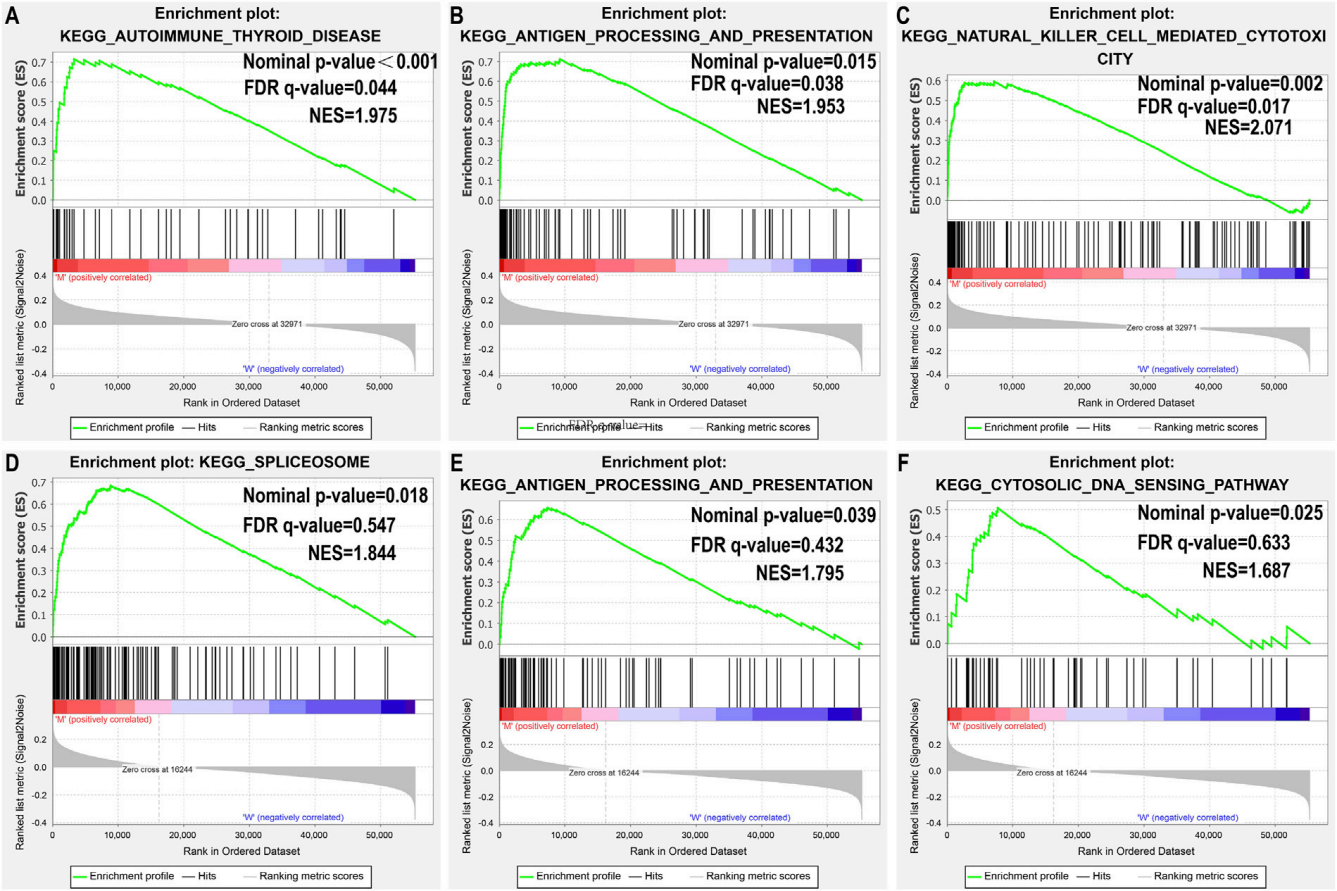
USH2A mutation is associated with immune-related pathways. Gene set enrichment analysis was performed with the TCGA. **(A–C)** Gene enrichment plots display that a series of immune-related gene sets are enriched in the USH2A-mutant group; **(D–F)** Gene enrichment plots display enrichment pathways in the MUC4-mutant group. The nominal *p*-value and FDR *q*-value is shown in each plot.

Since TMB has been reported as a biomarker for immunotherapy, and since *USH2A* and *MUC4* mutations are associated with increased TMB, we further studied the relationship between *USH2A*/*MUC4* mutations and immune response using TCGA data for GSEA. The *MUC4* mutation revealed no pathway with an FDR *q*-value < 0.05 (Figures 4D–F), whereas the *USH2A* mutation featured the following significantly upregulated pathways (Figures 4A–C): antigen processing and presentation pathways, thyroid autoimmune disease pathways, and NK cell-mediated cytotoxic pathways. These results indicate that the *USH2A* mutation affects the signaling pathways of the immune system.

### 3.4 *USH2A* Mutation in COAD is Associated With Tumor-Infiltrating Immune Cells

**FIGURE 5:**
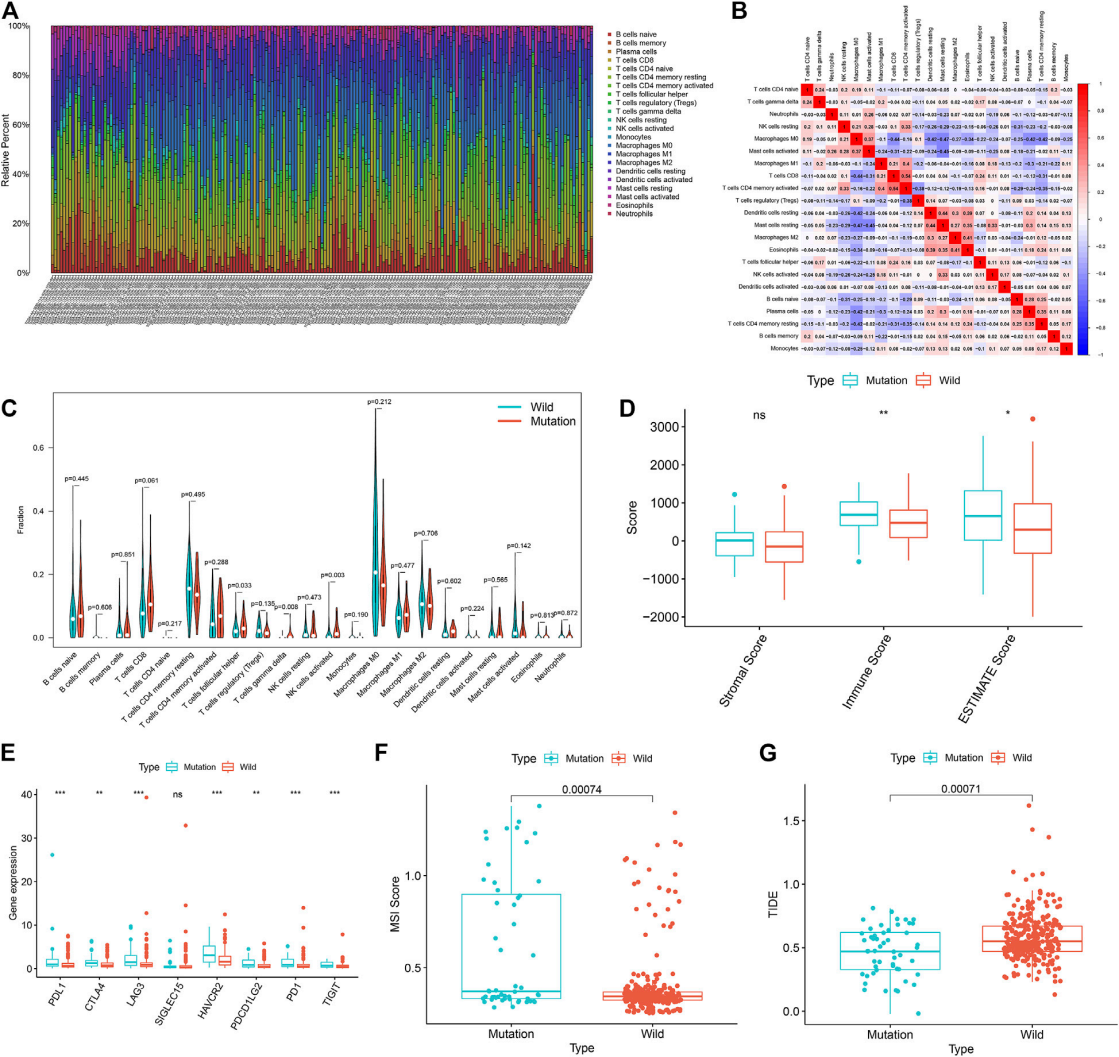
USH2A mutation is associated with tumor immune cell infiltration and antitumor immunity. **(A)** The stacked bar chart shows the distribution of 22 immune cells in each sample. **(B)** Correlation matrix of immune cell proportions. The red color represents a positive correlation, and the blue color represents a negative correlation. **(C)** Violin plot displaying the differentially infiltrated immune cells between the USH2A-mutant groups and the wild-type USH2A group. **(D)** Immune score of USH2A mutant group and USH2A wild group samples. **(E)** Gene expressions of eight common immune checkpoints in USH2A mutant group and USH2A wild group samples. **(F)** MSI scores of samples from the USH2A mutant group and USH2A wild group. **(G)** TIDE scores of USH2A mutant group and USH2A wild group samples. ***p < 0.001, **p < 0.01, *p < 0.05, ns has no statistical difference. Green represents the USH2A wild group, and red represents the USH2A mutant group.

GSEA results showed that the *USH2A* mutation affects the signaling pathways of the immune system. Therefore, we used the CIBERSORT algorithm to evaluate the relationship between the *USH2A* mutation and tumor-infiltrating immune cells in the colon cancer microenvironment. The results showed that the composition of 22 immune cells in each sample was significantly different (Figure 5A), and the immune score of *USH2A* mutation samples was significantly increased (Figure 5D). We also found that activated NK cells, follicular helper T cells (TFH cells), and *γδ*T cells were enriched in *USH2A* mutation samples (Figure 5C). In addition, the immune cell correlation matrix indicated activated NK cells, TFH cells, and *γδ*T, which were positively correlated with each other (Figure 5B).

### 3.5 *USH2A* Mutation Affects Immunotherapy

In cases with a *USH2A* mutation, among the common immune checkpoint genes (*PDL1*, *CTLA4*, *LAG3*, *SIGLEC15*, *HAVCR2*, *PDCD1LG2*, *PD1*, and *TIGIT*) (Wang et al., 2019a; Zeng et al., 2019), the expression levels of *PDL1*, *CTLA4*, *LAG3*, *HAVCR2*, *PD1*, *LG2*, and *TIGIT* were significantly increased (Figure 5E). We compared the MSI scores of the *USH2A* mutant group and the wild-type group, which showed that the MSI scores of the former were significantly increased (Figure 5F). TIDE uses a set of gene expression markers to estimate two different mechanisms of tumor immune evasion: tumor-infiltrating cytotoxic T lymphocyte (CTL) dysfunction and immunosuppressive factor rejection of CTL. A higher TIDE score denotes a higher chance of antitumor immune escape and a lower response rate of ICB therapy (Jiang et al., 2018). We compared the TIDE scores of the *USH2A* gene mutation group and the wild-type group, which showed that the TIDE score of the former was significantly reduced (Figure 5G). These results indicate that the *USH2A* mutation affects the tumor immune response and may lead to a better ICB treatment response.

### 3.6 Analysis of Differential Genes in Tumor Samples After *USH2A* Mutation and Constructing a Tumor Prognostic Risk Model Based on Differential Genes

**FIGURE 6:**
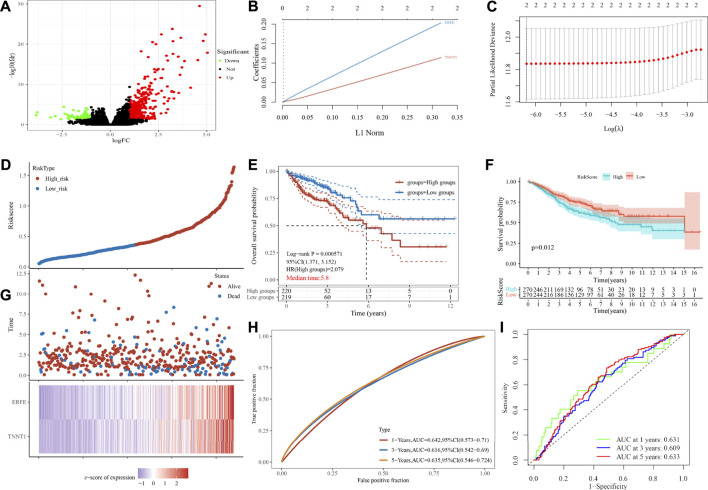
Analysis of gene differential expression after USH2A mutation and construction of a prognostic model based on the differentially expressed genes after USH2A mutation. **(A)** Volcano map of gene differential expression analysis. **(B)** Constructing the lasso coefficient prediction model. **(C)** Selecting variables in lasso regression with minimum criteria by 1,000 times cross-validation. **(D)**, **(G)** Risk plots for patients with higher and lower risk score. **(E)** Kaplan-Meier curve analysis of the high-risk and low-risk groups. **(H)** ROC curves of the risk score. **(F)** Kaplan-Meier curve analysis of the GSE39582 dataset based on risk model. **(I)** ROC curves of the risk score in the GSE39582 dataset based on risk model.

**FIGURE 7:**
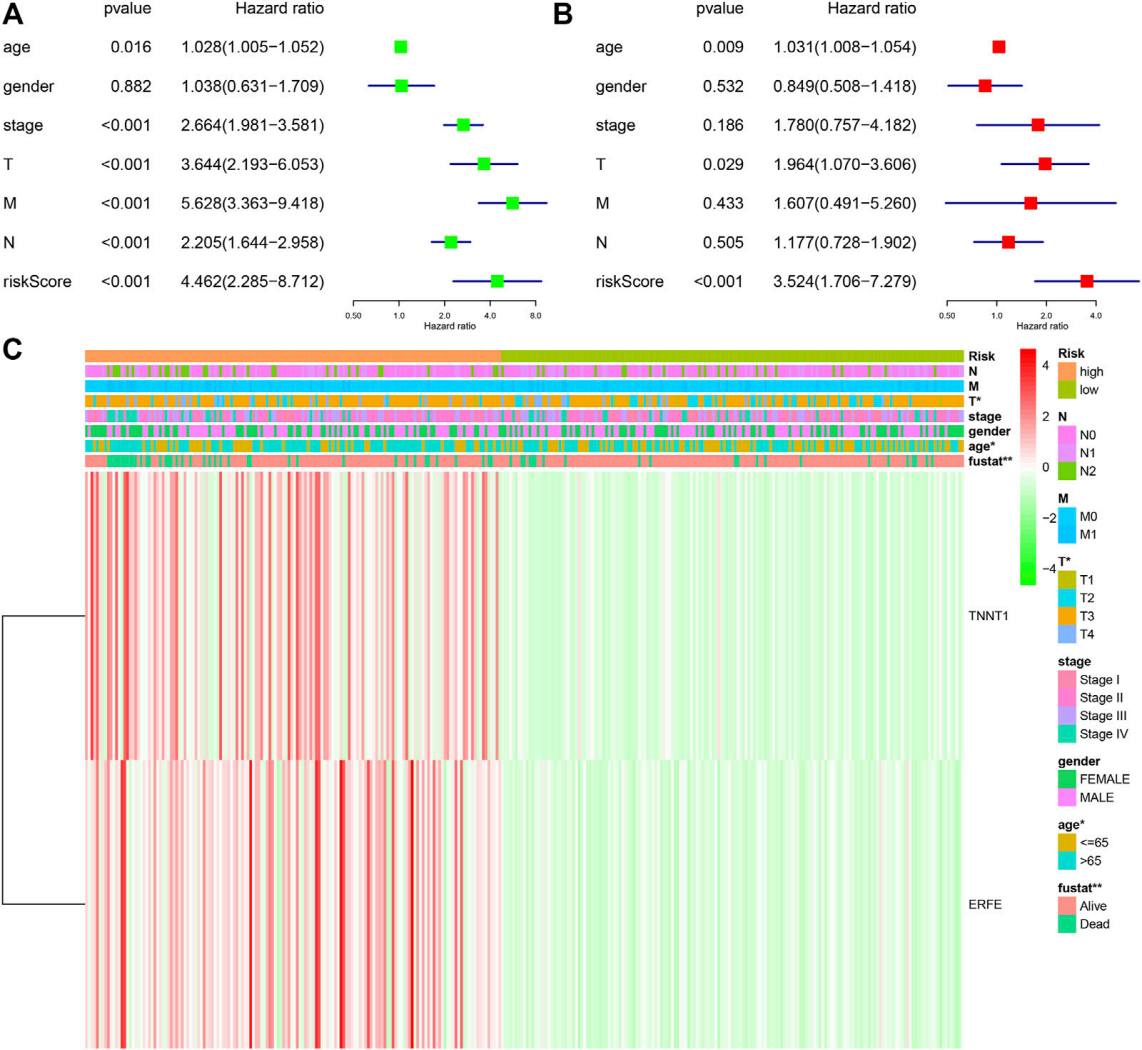
Correlation between risk model and clinical characteristics. **(A)** Univariate COX analysis forest plot based on TCGA samples. **(B)** Multivariate COX analysis forest plot based on TCGA samples. **(C)** Heat map of the correlation between risk scores and clinical characteristics in TCGA samples.

**FIGURE 8:**
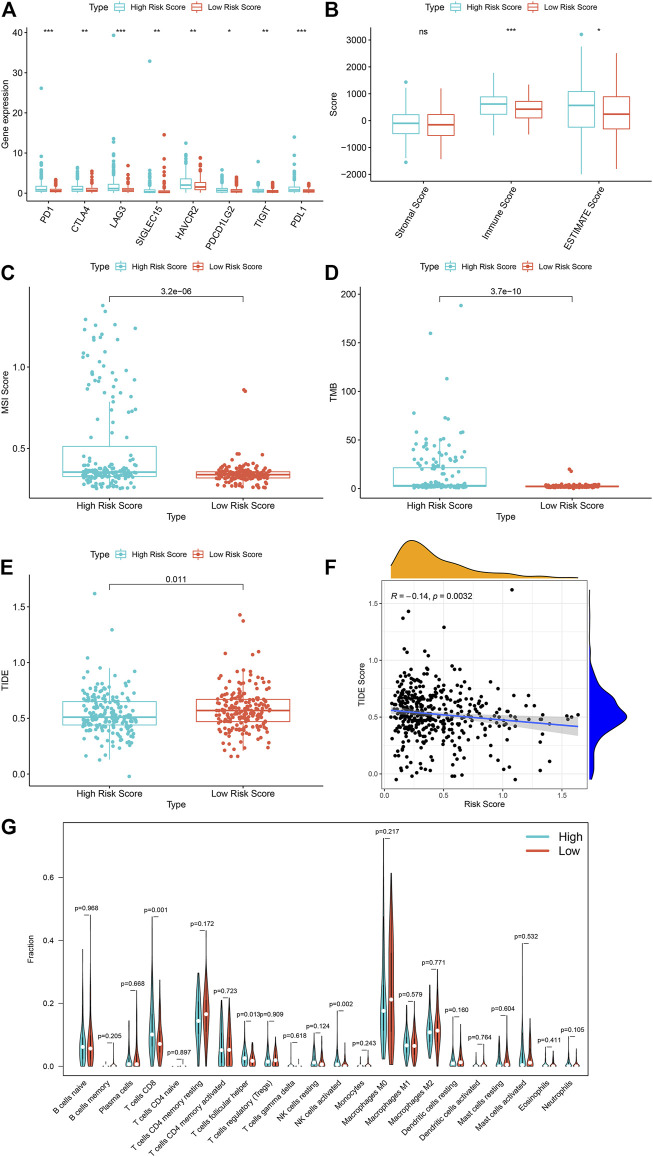
The high risk score is associated with a better tumor immunotherapy response in TCGA sample. **(A)** The expression of immune checkpoint genes in TCGA samples in high and low risk groups. **(B)** The immune scores of the high and low risk groups of TCGA samples. **(C)** The MSI scores of the high and low risk groups of the TCGA sample. **(D)** TMB situation of high and low risk groups of TCGA sample. **(E)** TIDE scores of the high and low risk groups of the TCGA sample. **(F)** Correlation analysis between TCGA sample risk score and TIDE. **(G)** Differences in immune cell infiltration between high and low model scores. ***p < 0.001, **p < 0.01, *p < 0.05, ns, has no statistical difference. Green represents the high-scoring group of the model, and red represents the low-scoring group of the model.

In order to further study the differential expression of tumor tissue genes after *USH2A* mutation, we divided TCGA COAD samples into a *USH2A* mutant group and a wild-type group, and we used the edgeR package to analyze the differential expression of genes. A total of 522 differentially expressed genes (DEGs) were obtained, among which 440 DEGs were upregulated and 82 DEGs were downregulated (Figure 6A). Univariate Cox analysis and LASSO COX analysis were performed on the above mentioned DEGs, and a prognostic risk model based on the expression of genes *TNNT1* and *ERFE* was established (lambda.min = 0.0021, RiskScore = (0.1141) × *TNNT1* + (0.2032 × *ERFE*) (Figures 6B–E,G). The ROC curve was drawn using R software package survival ROC (Figure 6H). We used the GEO database COAD dataset GSE39582 to validate the risk model (Figure 6F) and draw the ROC curve (Figure 6I). In the GSE39582 dataset, the survival of patients with high and low risk scores is significantly different (Figure 6F), indicating that the model has the ability to predict risk. Using the clinical data of TCGA COAD to test the correlation between the risk score and clinical characteristics, it was found that the age, survival status, and tumor T stage of patients were significantly different in the high- and low-risk groups (Figure 7C). Univariate and multivariate Cox analysis found that the risk score is an independent risk factor for the survival and prognosis of cancer patients in TCGA cohort (Figures 7A,B) and GSE39582 dataset (Supplementary Figure S1). Comparing the immune checkpoint gene expression, immune scores, MSI scores, TMB, and TIDE of patients in the high- and low-risk groups, we found significant differences (Figures 8A–E). We also compared the immune cell infiltration of samples from the high- and low-risk score groups, which showed that, in the high-risk group, CD8 T cells, TFH cells, and activated NK cells were significantly increased (Figure 8G). Our analysis found that the TIDE score was negatively correlated with the sample risk score (Figure 8F). These results indicate that patients at high risk with a poor survival prognosis may have a better response to ICB therapy, thereby improving their prognosis. Therefore, the risk model can predict the survival prognosis of cancer patients and guide the clinical treatment decisions of cancer patients.

## 4 Discussion

In summary, by analyzing the somatic mutation characteristics of 398 USA COAD samples in TCGA database and 305 Chinese COAD samples in the ICGC database, we found that *USH2A* is frequently mutated in both cohorts, and its mutation is associated with high TMB and poor clinical prognosis. We also found that the *USH2A* mutation is positively related to the signaling pathway of the immune system. The results of tumor-infiltrating immune cell analysis showed an enrichment of activated NK cells, TFH cells, and *γδ*T cells in the *USH2A* mutation samples, which is consistent with the results of previous studies (Bindea et al., 2013; Meraviglia et al., 2017; Zhang et al., 2020b). Dependent on the presence or absence of a *USH2A* mutation, we divided the TCGA COAD samples into two groups and analyzed the DEGs. According to the GO (Supplementary Figure S3A) and KEGG (Supplementary Figure S3B) enrichment analysis, we found that the DEGs mainly involved processes linked to cytokine activity and antibacterial humoral response and were significantly enriched in the IL-17 signaling pathway, which are all related to immune response. Querying the KEGG database (https://www.kegg.jp/), we found that NK cells and *γδ*T cells are involved in the IL-17 signaling pathway, which confirms that the pathway enrichment of DEGs after *USH2A* mutation is correlated with tumor immune cell infiltration. After *USH2A* mutation, analysis showed that immune checkpoint gene expression and TIDE score decreased significantly, whereas immune score and MSI score increased significantly, thus indicating that *USH2A* mutation affects the antitumor immunity and is conducive to ICB treatment. By performing univariate Cox analysis and LASSO COX analysis of DEGs, we established a prognostic risk model based on the expression of genes *TNNT1* and *ERFE*. Cox analysis showed that risk score is an independent risk factor for tumor survival and prognosis. The verification of the ROC curve using the GSE39582 dataset showed that the model has the ability to predict risk. Comparing the immune checkpoint gene expression, immune score, MSI score, TMB, and TIDE of patients in the high- and low-risk groups, significant differences were found, whereby CD8 T cells, TFH cells, and activated NK cells were all significantly increased in the high-risk group. These results indicate that the risk model can predict the survival prognosis of COAD patients and assess whether the patients will have a good ICB treatment response.

The *USH2A* (also known as Usherin) gene encodes a protein. The protein exists in the basement membrane and may play an important role in the development and homeostasis of the inner ear and retina (Weston et al., 2000). *USH2A* mutations are associated with Usher syndrome type IIa, retinitis pigmentosa (Xing et al., 2020), and tongue squamous cell carcinoma (Zhang et al., 2020). In lung adenocarcinoma, the *USH2A* mutation is one of the most frequently mutated genes for predicting neoantigens (Cai et al., 2018). In our research, we found that *USH2A* mutation is associated with the overexpression of immune checkpoint genes and increased TMB. TMB represents the accumulation of somatic mutations in tumors. A high TMB helps to expose more neoantigens, which may trigger a T-cell-dependent immune response (Mcgranahan et al., 2016). Immune checkpoint blockade (ICB), which targets programmed cell death ligand 1 (*PDL1*) and cytotoxic T lymphocyte antigen 4 (*CTLA4*) pathways, has become a treatment strategy for various types of cancer (Long et al., 2017; Zhang et al., 2021). We used TCGA dataset to analyze the tumor response to immunotherapy after *USH2A* mutation. We found that *USH2A* mutant tumors have stronger immunogenicity, exhibited as higher TMB, increased immune cell infiltration into tumor tissues, and overexpression of immune checkpoint factors such as *PD1*, *PDL1*, and *CTLA4*. This indicates that the *USH2A* mutation can enhance tumor immunogenicity, allowing tumor patients to benefit from antitumor immunotherapy. The expression of *PDL1* and TMB are correlated with the clinical benefit of patients treated with ICB (Long et al., 2017). However, these two biomarkers are continuous variables with no clearly defined cutoff point above which a response is guaranteed. In addition, the expression of *PDL1* and TMB also vary depending on the detection method and platform (Tsao et al., 2018; Addeo et al., 2019). In contrast, *USH2A* mutations are easily detected by next-generation sequencing, and their presence in this study was closely related to the response to ICB treatment. Therefore, it is worth considering the *USH2A* mutation as a potential biomarker for the sensitization of patients to ICB therapy.

NK cells play a key role in innate and adaptive immune response and tumor immune surveillance by recognizing and killing tumor cells (Mandal and Viswanathan, 2015). Although tumor-related NK cells are not common in tumor immune infiltration, they have been associated with increased survival of colon cancer patients (Melero et al., 2014). Higher NK cell activity is associated with poor prognosis of skin T cell lymphoma (Mundy-Bosse et al., 2018). Explanations for this difference include the impaired recognition of malignant CD4^+^ T cells mediated by NK cells and the inability of NK cells to form functional immune synapses (Mundy-Bosse et al., 2018). TFH cells are specialized T helper cells, and their most significant role is to promote the formation and maintenance of germinal centers, as well as the maturation of B cells and the acquisition of immune memory (Vinuesa et al., 2016). Currently, it is generally believed that the TFH cell–B cell axis in tumor-associated tertiary lymphoid structures (TLSs) is conducive to the formation of an antitumor immune environment (Galon et al., 2013). TFH cells produce chemokine ligand 13 (CXCL13), which targets B cells and TFH cells themselves *via* chemokine receptor 5 (CXCR5). High numbers of TFH cells and high levels of CXCL13 are associated with increased survival of colon cancer patients (Bindea et al., 2013). *γδ* T cells in the colon are the first line of defense against pathogens in the intestinal tissue immune monitoring program (Suzuki et al., 2020). Evidence has shown that human *γδ* T cells have antitumor effects in colon cancer, which is related to their ability to kill established colon cancer cells (Suzuki et al., 2020). The knowledge of how *γδ* T cells promote colon cancer is still limited, but the *γδ* T cells known to promote the progression of colon cancer are mainly concentrated in the *γδ* T cell subset that produces IL-17 (Van hede et al., 2017). In breast tumors and gallbladder tumors, an increase in *γδ* T cells is associated with poor prognosis (Ma et al., 2012; Patil et al., 2016). There has not been a comprehensive histological analysis of the prognostic ability of *γδ* T cells in colon cancer. In a follow-up study of colon cancer patients, the immune score (including tumor-infiltrating *γδ* T cells) was used to group patients. The 5 years recurrence rate of patients in the high-immune-score group was only 4.8% (Pages et al., 2009). Considering the relationship between high immune score and good prognosis, it has been speculated that *γδ* T cells may be associated with a better colon cancer prognosis (Suzuki et al., 2020). In this study, the survival prognosis of patients with *USH2A* mutations was poor; however, in the *USH2A* mutant tumor samples, there was an enrichment of activated NK cells, TFH cells, and *γδ* T cells, indicating a change in the recognition of immune surveillance, as well as an antitumor effect. Therefore, we found that, in COAD, the *USH2A* mutation can induce changes in infiltrating immune cells, thereby enhancing antitumor immunity.

The increased migration and invasion potential of colon cancer cells leads to a significant decrease in the 5 years survival rate of colon cancer patients. Therefore, an accurate prediction of prognosis is essential for individualized treatment of these patients. Today, gene expression profiling has become an adjunct to cancer treatment; for example, Gene-expression prediction models were built using transcriptome to predicte colorectal cancer risk (Guo et al., 2021), the expression characteristics of six lncRNAs were used as indicators to evaluate the prognosis of patients with colorectal cancer (Zhao et al., 2018), and an eleven gene signature was used as prognostic index to predict systemic recurrences in colorectal cancer (Kim et al., 2019). In this study, we identified the expression levels of two mRNAs as reliable prognostic indicators of colon cancer. In this risk model, the *TNNT1* gene encodes a protein of the troponin subunit, which is a regulatory complex located on the sarcomere filaments (Wei and Jin, 2016). Studies have reported that *TNNT1* is significantly upregulated in colon cancer samples and cell lines. The upregulation of *TNNT1* is also related to a variety of clinicopathological characteristics, and its high expression is related to the poor prognosis of patients. Inhibition of *TNNT1* can significantly inhibit cell proliferation, migration, and invasion, while promoting cell apoptosis (Chen et al., 2020). *TNNT1* may promote the progress of COAD and mediate the EMT process (Hao et al., 2020). On the other hand, ERFE is a glycoprotein hormone encoded by *FAM132B*, which is produced upon the stimulation of red blood cells by erythropoietin in the bone marrow and spleen (Ganz, 2019). It has been reported that this gene is mainly related to anemia and metabolic abnormalities (Seldin et al., 2012; Bondu et al., 2019), whereas there are no reports of a tumor connection. *TNNTI* gene expression in our research model was associated with poor tumor prognosis, which is consistent with previous studies (Hao et al., 2020). Patients with a higher TIDE score have a higher chance of antitumor immune escape, thus showing a lower response rate to ICB therapy (Jiang et al., 2018). In our study, a comparison of the TIDE of patients in the high-risk and low-risk groups revealed a lower score in the former, which indicates the potential for high-risk patients to improve their survival prognosis through a better response to ICB therapy, which can facilitate the choice of clinical treatment for cancer patients.

The novelty of this study lies in the discovery that *USH2A* mutations can affect the antitumor immunity of COAD and the responsiveness to ICB therapy. Furthermore, we constructed a prognostic model consisting of two DEGs, which could predict 1, 3, and 5 years survival rates in TCGA dataset and GEO validation dataset GSE39582 with a relatively high AUC. The main limitation of this study is that the ICGC database lacks corresponding clinical data on Chinese COAD; thus, we could not verify the significance of the *USH2A* mutation in the prognosis of Chinese COAD patients and whether it can cause the same immune response. Even though *USH2A* was frequently mutated in Chinese COAD samples, its impact may be somewhat heterogeneous among different races. Therefore, the relationship between *USH2A* mutation and prognosis, including the analysis of infiltrating immune cells and signaling pathways, needs to be further verified in Chinese colon samples. In addition, the differential expression of the two genes used to construct the risk model was identified from TCGA data; although TCGA data are of high quality, further experimental verification of the role of these two differential genes in colon cancer is needed *in vitro* and *in vivo*.

In summary, this study showed that *USH2A* is frequently mutated in COAD, which is associated with a high TMB and poor prognosis. In addition, the *USH2A* mutation upregulates immune signaling pathways and promotes an antitumor immune response. On the basis of two DEGs associated with the *USH2A* mutation, we constructed a model with a predictive effect on the prognosis of tumor survival. These findings reveal a new gene whose mutation can be used as a biomarker for predicting the response to antitumor immunity and ICB treatment.

## Data Availability

The datasets presented in this study can be found in online repositories. The names of the repository/repositories and accession number(s) can be found in the article/Supplementary Material.

## Supplementary Material

Supplementary Figure 1 Riskscore is an independent prognostic factor in the validation data set GSE39582. **(A)** Univariate COX analysis forest plot based on GSE39582 dataset. **(B)** Multivariate COX analysis forest plot based on GSE39582 dataset.

Supplementary Figure 2 The relationship between the location of the USH2A mutation site and the survival of COAD in the COAD sample of the TCGA database. Kaplan-Meier survival analysis was used to determine survival curves that reflect the association between gene mutations in exons 17, 61, 63, 64, 70 and prognosis.

Supplementary Figure 3 DAVID was used for GO enrichment and KEGG pathway analysis of DEGs. **(A)** Analysis of GO enrichment of differentially expression genes. **(B)** Analysis of GO enrichment of differentially expression genes.
