# Supplementary figures and images for: USH2A Mutation is Associated With Tumor Mutation Burden and Antitumor Immunity in Patients With Colon Adenocarcinoma
Source: Front Genet. 2021 Nov 2;12:762160. doi: 10.3389/fgene.2021.762160 (PMC8593250; doi:10.3389/fgene.2021.762160)

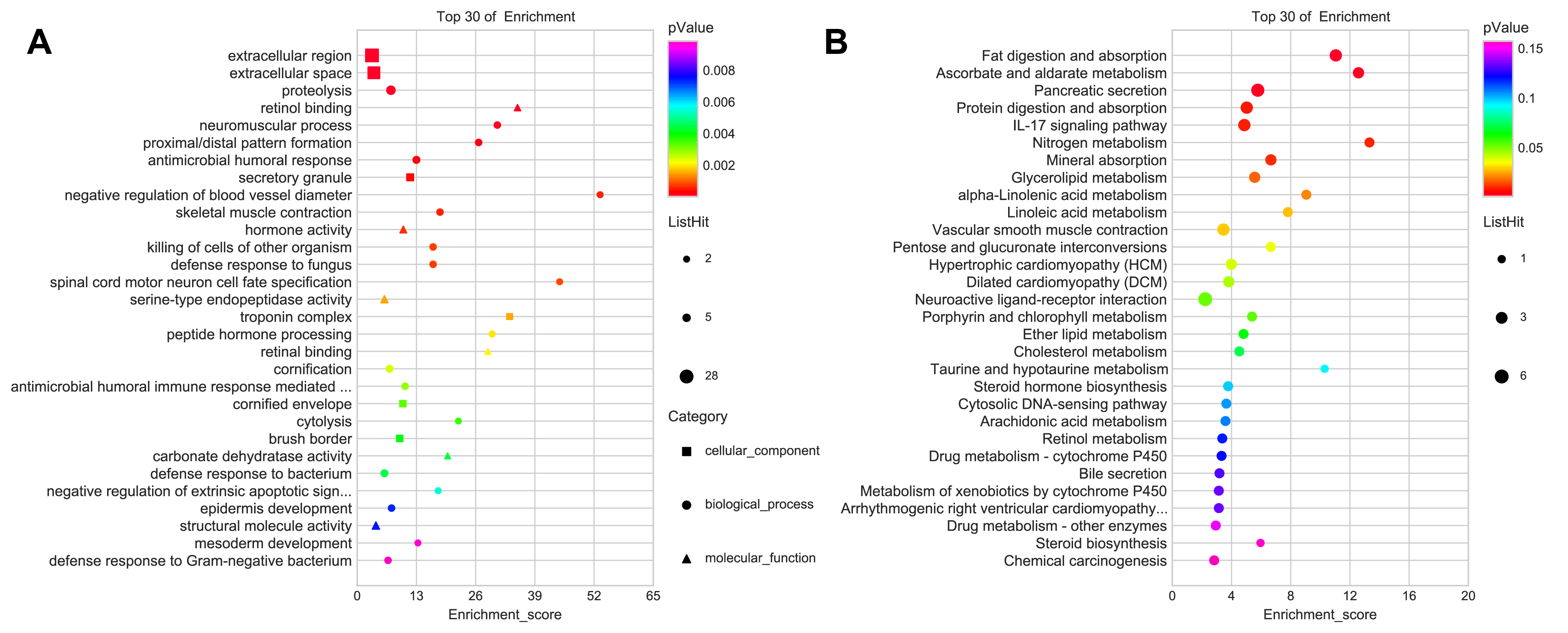

Supplement: Supplementary file 1 [file Image3.tif]

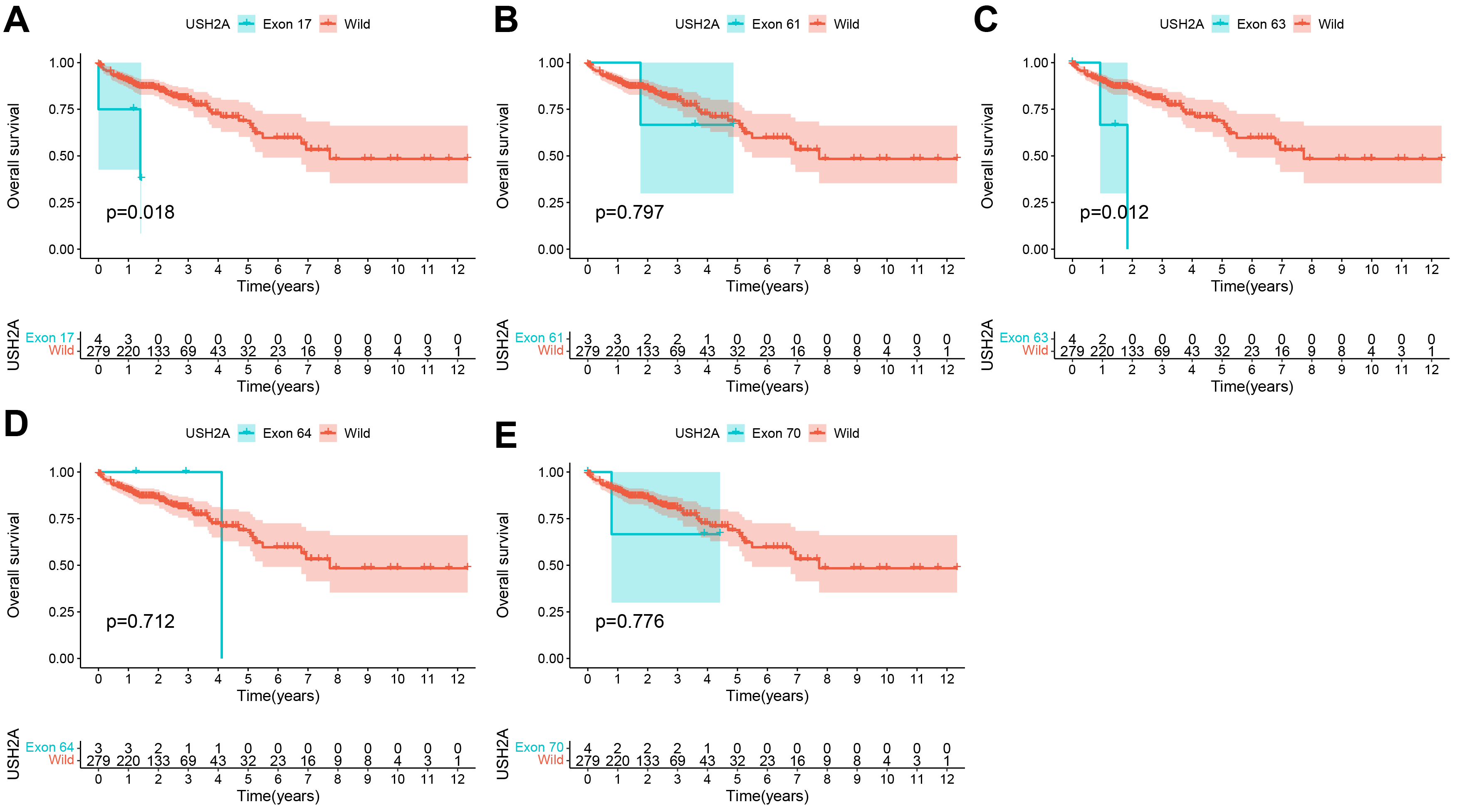

Supplement: Supplementary file 2 [file Image2.tif]

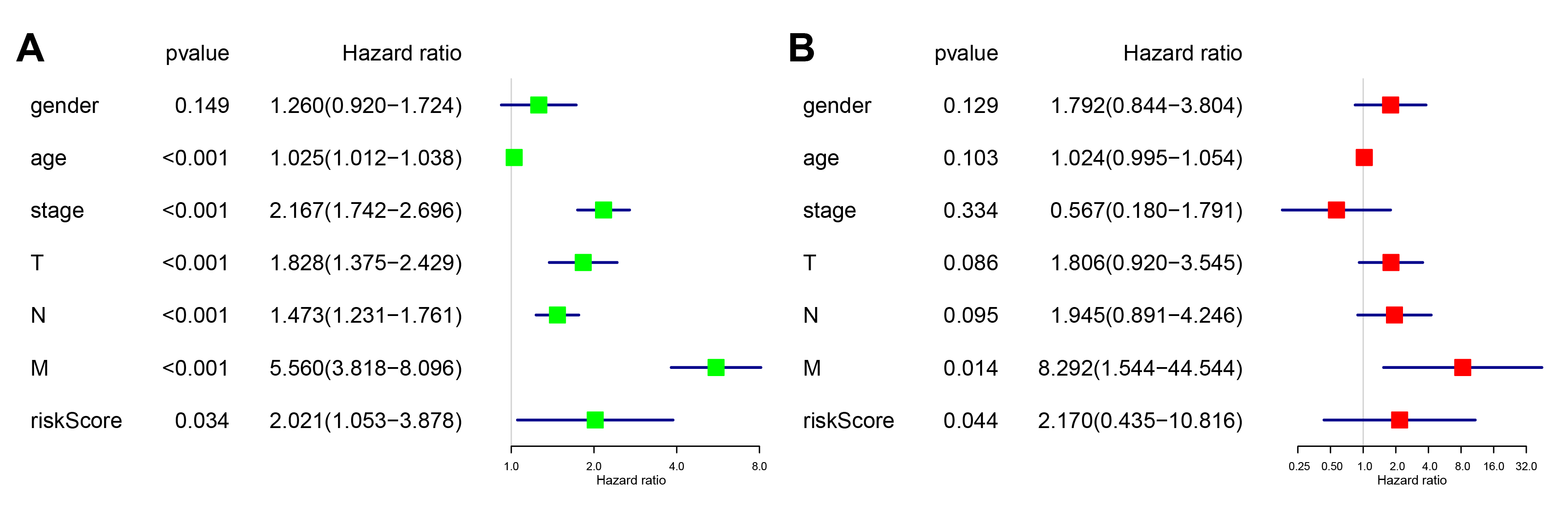

Supplement: Supplementary file 3 [file Image1.tif]
