# Supplementary material for: USH2A Mutation is Associated With Tumor Mutation Burden and Antitumor Immunity in Patients With Colon Adenocarcinoma
Source: Front Genet. 2021 Nov 2;12:762160. doi: 10.3389/fgene.2021.762160 (PMC8593250; doi:10.3389/fgene.2021.762160)
Supplement: Supplementary file 4 [file DataSheet1.docx]

| Gene | Mutation sample | Wild sample | Gene | Mutation sample | Wild sample |
| --- | --- | --- | --- | --- | --- |
| APC | 249 | 81 | FAT3 | 49 | 281 |
| TP53 | 192 | 138 | RYR2 | 59 | 271 |
| TTN | 153 | 177 | PIK3CA | 89 | 241 |
| MUC16 | 86 | 244 | FBXW7 | 50 | 280 |
| MUC4 | 47 | 283 | DNAH11 | 52 | 278 |
| SYNE1 | 91 | 239 | MUC5B | 42 | 288 |
| FLG | 48 | 282 | ZFHX4 | 68 | 262 |
| FAT4 | 74 | 256 | NEB | 44 | 286 |
| PCLO | 54 | 276 | USH2A | 51 | 279 |
| OBSCN | 64 | 266 | LRP1B | 54 | 276 |
| CSMD1 | 52 | 278 |  |  |  |

Table S1. The mutation frequency of 21 genes related to tumor mutation burden in TCGA samples.

Table S2.The mutation region of USH2A in 51 COAD samples.

| TCGA ID | Mutation region of USH2A | TCGA ID | Mutation region of USH2A | TCGA ID | Mutation region of USH2A |
| --- | --- | --- | --- | --- | --- |
| TCGA-CA-6718 | Exon 72 | TCGA-AZ-4315 | Exon 61 | TCGA-AA-A004 | Exon 25 |
| TCGA-A6-2686 | Exon 71 | TCGA-CK-5916 | Exon 61 | TCGA-SS-A7HO | Exon 24 |
| TCGA-5M-AAT6 | Exon 70 | TCGA-F4-6807 | Exon 61 | TCGA-A6-2679 | Exon 23 |
| TCGA-A6-2672 | Exon 70 | TCGA-F4-6570 | Exon 60 | TCGA-AA-3663 | Exon 23 |
| TCGA-AA-3864 | Exon 70 | TCGA-AA-3977 | Exon 55 | TCGA-QL-A97D | Exon 18 |
| TCGA-NH-A5IV | Exon 70 | TCGA-F4-6855 | Exon 53 | TCGA-AA-3492 | Exon 17 |
| TCGA-AM-5821 | Exon 69 | TCGA-CM-6161 | Exon 52 | TCGA-CM-6171 | Exon 17 |
| TCGA-CM-4746 | Exon 68 | TCGA-AA-3855 | Exon 50 | TCGA-D5-6538 | Exon 17 |
| TCGA-AA-3511 | Exon 66 | TCGA-G4-6320 | Exon 45 | TCGA-NH-A8F8 | Exon 17 |
| TCGA-AA-3984 | Exon 65 | TCGA-AA-3952 | Exon 44 | TCGA-5M-AAT4 | Exon 13 |
| TCGA-AA-A010 | Exon 64 | TCGA-G4-6586 | Exon 42 | TCGA-A6-5660 | Exon 11 |
| TCGA-AZ-6598 | Exon 64 | TCGA-D5-5538 | Exon 41 | TCGA-DM-A1D8 | Exon 9 |
| TCGA-CM-5861 | Exon 64 | TCGA-A6-3810 | Exon 37 | TCGA-AZ-6601 | Exon 8 |
| TCGA-AA-3672 | Exon 63 | TCGA-AA-A02H | Exon 36 | TCGA-AA-3861 | Exon 6 |
| TCGA-AA-3846 | Exon 63 | TCGA-AA-3489 | Exon 35 | TCGA-AD-6964 | Exon 6 |
| TCGA-AA-A02R | Exon 63 | TCGA-AA-3510 | Exon 35 | TCGA-G4-6311 | Exon 5 |
| TCGA-F4-6461 | Exon 63 | TCGA-AY-5543 | Exon 30 | TCGA-AD-6895 | Exon 3 |
